# The regulatory network of *E. coli *metabolism as a Boolean dynamical system exhibits both homeostasis and flexibility of response

**DOI:** 10.1186/1752-0509-2-21

**Published:** 2008-02-29

**Authors:** Areejit Samal, Sanjay Jain

**Affiliations:** 1 Department of Physics and Astrophysics, University of Delhi, Delhi 110007, India; 2 Jawaharlal Nehru Centre for Advanced Scientific Research, Bangalore 560064, India; 3 Santa Fe Institute, 1399 Hyde Park Road, Santa Fe, NM 87501, USA

## Abstract

**Background:**

Elucidating the architecture and dynamics of large scale genetic regulatory networks of cells is an important goal in systems biology. We study the system level dynamical properties of the genetic network of *Escherichia coli *that regulates its metabolism, and show how its design leads to biologically useful cellular properties. Our study uses the database (Covert *et al*., Nature 2004) containing 583 genes and 96 external metabolites which describes not only the network connections but also the Boolean rule at each gene node that controls the switching on or off of the gene as a function of its inputs.

**Results:**

We have studied how the attractors of the Boolean dynamical system constructed from this database depend on the initial condition of the genes and on various environmental conditions corresponding to buffered minimal media. We find that the system exhibits homeostasis in that its attractors, that turn out to be fixed points or low period cycles, are highly insensitive to initial conditions or perturbations of gene configurations for any given fixed environment. At the same time the attractors show a wide variation when external media are varied implying that the system mounts a highly flexible response to changed environmental conditions. The regulatory dynamics acts to enhance the cellular growth rate under changed media.

**Conclusion:**

Our study shows that the reconstructed genetic network regulating metabolism in *E. coli *is hierarchical, modular, and largely acyclic, with environmental variables controlling the root of the hierarchy. This architecture makes the cell highly robust to perturbations of gene configurations as well as highly responsive to environmental changes. The twin properties of homeostasis and response flexibility are achieved by this dynamical system even though it is not close to the edge of chaos.

## Background

Large scale biological networks and their associated dynamical systems have a crucial role to play in unravelling the systemic properties of cells. Structural studies of large scale metabolic, protein interaction and genetic regulatory networks have uncovered some unexpected patterns leading to interesting hypotheses and questions (for reviews see [1-3]). For a deeper understanding of system level phenomena, it now seems that we need to explore the relationship between network structure and the dynamics of genes, proteins and other biomolecules. In this paper we study the *Escherichia coli *regulatory network and show that the dynamics leads to biologically important properties such as cellular homeostasis and flexibility of response to varied environments. Our study reveals that some very simple features of the genetic regulatory network are responsible for these properties. These design features may be universal across prokaryotes and possibly have vestiges in higher organisms as well.

Large scale mathematical models for dynamical phenomena are difficult to construct due to paucity of data and are difficult to profitably analyze due to their complexity. In this context flux balance analysis (FBA) has proved to be a useful computational technique to explore steady state flows in large scale metabolic networks [4-7]. A conceptual framework to study dynamics of large scale genetic regulatory networks as Boolean systems was introduced by Kauffman [8-10]. In this paper we use this approach to study the large scale transcriptional regulatory network (TRN) of an organism in which both the network and the Boolean functions have been constructed from real data. Our study is based on the database iMC1010v1 [11] which describes the regulatory network controlling metabolism in *E. coli*.

The Boolean approach provides a coarse-grained model of the dynamics of TRNs, in which each gene's configuration has only two allowed values (corresponding to the gene being off or on), each gene's update is given by a Boolean function of all its inputs, time is discrete and (in our work) all genes are updated synchronously. A differential equation based simulation of large scale TRNs is not feasible at the moment due to lack of kinetic data, and the large number of unknown parameters would also render the results of such a simulation difficult to interpret [12]. On the other hand Boolean simulations of smaller biological systems have provided useful insights [13-17]. The Boolean approach can provide useful information about some qualitative features of the dynamics, e.g., the nature of the attractors of the system, and through that, insights about what might happen in a more detailed simulation and the system itself.

### The genetic network regulating *E. coli *metabolism as a Boolean dynamical system

**Figure 1:**
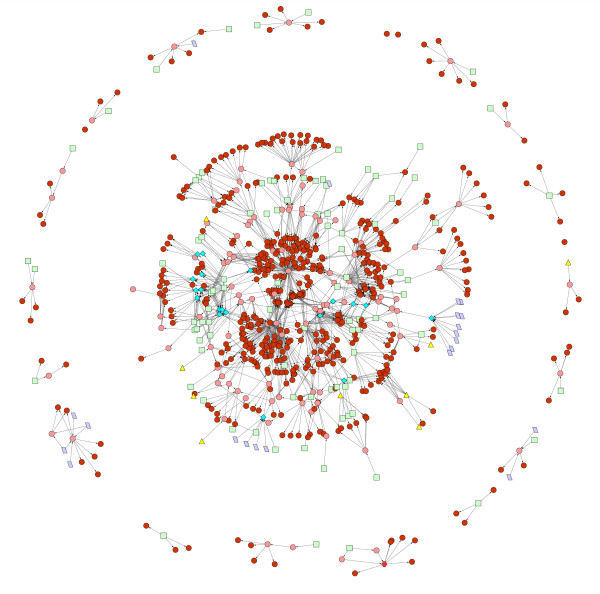
Map of the transcriptional regulatory network controlling metabolism in *E. coli*. In this figure, there are genes coding for the TFs (pink circles), genes coding for enzymes (brown circles), external metabolites (green squares), certain internal fluxes (purple parallelograms), stimuli (yellow triangles) and other conditions (blue diamonds). See text for details.

The database iMC1010v1 contains 583 genes. These are collectively regulated by a set of 103 transcription factors (TFs) which are gene products of 104 of the genes in the set, 96 external metabolites, 19 other conditions, 21 internal fluxes of metabolic reactions and 9 stimuli. The directed graph of this network is shown in Fig. 1, where a directed link from one node to another denotes a regulatory interaction.

The database also provides the Boolean input-output map at each node, e.g., the configuration of each gene (on or off), as a function of the on-off states of all its inputs. Using this information we construct the following discrete dynamical system describing *E. coli's *TRN (for details, see Methods section):

*g*_*i*_(*t *+ 1) = *G*_*i*_(**g**(*t*), **m**);   *i *= 1, 2,..., 583.

Here *g*_*i*_(*t*) is the configuration of gene *i *at time *t*. Time is measured in discrete units: *t *= 0, 1, 2,...* g*_*i*_(*t*) = 1 (0) means that at time *t *gene *i *is on (off). The vector **g**(*t*) collectively denotes the configurations of all the genes at time *t*; its *i*^*th *^component is *g*_*i*_(*t*). The vector **m **denotes the configuration of external metabolites; its *i*^*th *^component *m*_*i *_= 1 if metabolite *i *(*i *= 1, 2,...,96) is present in the external environment for uptake into the cell, and *m*_*i *_= 0 if it is absent. The above equation expresses the fact that the on-off state of a gene at any time instant is controlled by the state of the genes at the previous time instant as well as the state of the external environment. The interaction of genes is mediated by transcription factors. Thus a single time unit corresponds to the average time between the initiation of transcription of a gene coding for a transcription factor and the initiation of transcription of a gene regulated by that transcription factor.

In principle **m **can also change with time as the cell uses up food molecules in its external environment for its metabolism and excretes other molecules [18,19]. However, in the present work we consider only buffered media which are characterized by *m*_*i *_that are constant in time. **m **thus defines a constant external environment of the cell. We have considered two classes of buffered media, (a) a set of 93 minimal media (62 aerobic and 31 anaerobic) each capable of supporting the growth of the cell as determined by FBA (see Supplementary Table S1 in Additional File 1 for a list), and (b) a much larger library of 109732 minimal media constructed using the method described by Barrett et al [19].

**Figure 2:**
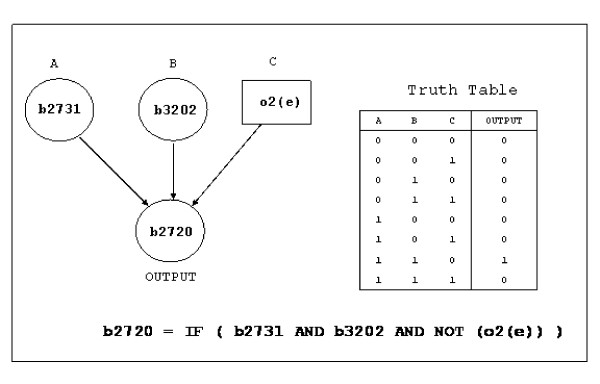
Example of a Boolean function *G*_*i *_representing the regulatory logic at the promoter region of gene b2720 that determines its expression. The gene b2720 is on if and only if both the transcription factors coded by genes b2731 and b3202 are present and oxygen is absent in the environment. For all other cases, the gene b2720 is off.

The functions *G*_*i *_contain all information about the internal wiring of the network (who influences whom) as well as the logic of each gene's regulation (given the configuration of all of gene *i*'s inputs at time *t*, whether gene *i *will be on or off at *t *+ 1). Each function *G*_*i *_typically depends only upon those components of **g **and **m **that directly affect the expression of gene *i *(see Fig. 2 for an example). We have considered the dynamical system (1) with two slightly different forms of the functions *G*_*i*_, called 1A and 1B, arising from two different treatments of intermediate variables (the internal fluxes of certain metabolic reactions) that appear in the database iMC1010v1. In the first approach (1A) for simplicity we have treated only the genes and their products as dynamical variables, keeping these internal fluxes fixed. The second approach (1B) includes the effect of some other internal variables such as concentrations of internal metabolites (as reflected through these fluxes) also being dynamical. The latter effectively introduce additional interactions among the genes.

The conceptual framework for studying TRNs as Boolean dynamical systems of the type *g*_*i*_(*t *+ 1) = *G*_*i*_(**g**(*t*)) was set up by Kauffman [8,9] almost four decades back and subsequently has been studied extensively, resulting in several important insights [10,20-24]. In particular Kauffman found that such systems with a large number of components possess an ordered regime in which the attractors have short periods and large basins. In this regime these systems have the property of homeostasis or robustness to perturbations of the genetic configuration. In the absence of detailed molecular data on the real genetic networks, this approach was used for ensembles of biologically motivated random Boolean networks, and, more recently, real networks with the functions *G*_*i *_chosen randomly from a suitable ensemble of Boolean functions [23,24].

References [13-17] have applied the Boolean approach to specific biological gene regulatory networks where detailed genetic data is available. These networks are smaller than the ones mentioned above, and have up to 40 distinct genes, proteins and other molecules [13-17]. In reference [14], where a Boolean network of 180 nodes is considered, the network contains 15 distinct genes and proteins (with 12 nodes for each of them corresponding to 12 distinct cells). These models, apart from reproducing several observed phenomena of these systems, have also found that the networks possess the property of homeostasis, as well as robustness to genetic mutations.

The present study is inspired by the work of Kauffman and extends the above development in two important ways. One, it studies the empirically derived network of a real organism, but one that is much larger than the biological systems mentioned above. The present network [11] has 583 genes and 96 external metabolites accounting for close to half of all genes currently believed to be involved in metabolism in *E. coli*. Being more than an order of magnitude larger (in terms of the number of genes involved) than other real genetic networks considered as Boolean systems, this allows us a qualitatively different systemic view of the organization of the genetic network of an organism. We not only find homeostasis in this large system, but also identify the design feature of the network responsible for this property. Two, we are able to study the effect of the external environment on the TRN dynamics through the vector **m **in Eq. (1). Note that the system studied by Kauffman is described by the equation *g*_*i*_(*t *+ 1) = *G*_*i*_(**g**(*t*)) instead of Eq. (1), which takes into account the effect of genes on other genes but not the effect of the external environment. Other works that investigate real biological systems as Boolean networks have only a few environmental signals [13-17]. As a consequence of the present database [11], we are able to take into account the effect of external environment in a much more systematic and extensive way than before. This sheds light on a different property of the network, namely its flexibility of response to a diversity of environments.

## Results

### Homeostasis: The final state is essentially the same after any perturbation of the genes

**Figure 3:**
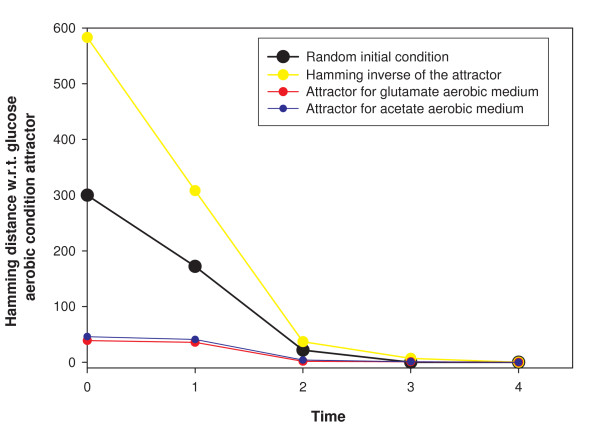
Dynamical behaviour of the *E. coli *TRN for a fixed environment, glucose aerobic minimal media. For all initial conditions the system is attracted to a fixed point whose configuration depends upon the medium. The plots depict, as a function of time, the hamming distance of the configuration from the fixed point attractor corresponding to the medium. 4 different initial conditions are shown. One is a randomly chosen initial condition. Another is the 'hamming inverse' of the attractor (in which the configuration of every gene is reversed with respect to the attractor). Two other initial conditions are the attractor configurations of other minimal media.

We simulated the dynamical system 1A for each of the 93 **m **vectors corresponding to the 93 minimal media mentioned above, starting from a set of 10000 randomly chosen initial conditions for the *g*_*i*_. For each **m **and each initial condition of the genes, the system reached a fixed point attractor in a maximum of 4 time steps. Furthermore, for each **m **the fixed point was independent of the chosen initial condition of the genes. This is shown in Fig. 3 for glucose aerobic medium for four initial conditions. We also considered the library of 109732 minimal media for a single randomly chosen initial condition each. A fixed point attractor was found in each case. There are in principle 2^583 ^possible initial conditions. We present later the analytic argument as to why a unique final configuration independent of initial condition is inevitable for each fixed **m**, given the architecture of the TRN. This property means that as long as the external environment remains fixed, the TRN regulating *E. coli *metabolism will revert to a unique configuration of its genes after any perturbation of the latter.

The dynamical system 1B, which includes some additional links between the genes compared to 1A, was also studied for the 93 minimal media with 1000 randomly chosen initial conditions each. In this case for 89 of the 93 media, we found 36 distinct attractors (8 fixed point attractors and 28 two-cycles). For the remaining 4 minimal media, there were 10 distinct attractors (4 fixed point attractors and 6 two-cycles). Again the attractor was reached in a maximum of 4 time steps. For each of the cycles, we found that most of the genes (562 to 567 out of 583) were in fact frozen in a fixed configuration, and only 16 to 21 genes oscillated back and forth between zero and one with period two. These 21 genes are listed in Supplementary Table S2 in Additional File 1. Furthermore for any given medium we found that each of the 562 frozen genes had the same configuration across all the attractors (36 or 10). This means that for any given medium, most genes (562 or more out of 583) end up in the same fixed configuration independent of the initial conditions of the genes. Recently, Shlomi *et al*. [25], using a different technique, also observed that the state of only 10 genes is undetermined for a given medium in the regulatory network controlling *E. coli *metabolism [11]. One can show that there are no other attractors of system 1B, using its structural properties (analysis not presented here). We have also checked that the 562 frozen genes end up in the same configuration in both system 1A and 1B for any given medium.

Kauffman has characterized random Boolean networks as having two regimes, an ordered regime wherein the attractors have a large 'frozen core' of genes locked in a fixed configuration together with a few 'twinkling islands' of genes that switch on and off, and a chaotic regime wherein the number of 'frozen' genes is much less than those of the 'twinkling' ones [10]. Our findings above imply that the genetic regulatory network controlling *E. coli*'s metabolism is deep in the ordered regime, since the dynamical variables corresponding to 562 out of 583 genes are frozen in a fixed configuration when the external environment is fixed. Collectively, our results of both dynamical systems imply that the *E. coli *TRN exhibits a high degree of homeostasis, in that it is highly insensitive to initial conditions and for any given medium all genetic perturbations die out quickly, restoring an overwhelming majority of genes to a configuration that is independent of the perturbation.

### Flexibility: The system has a wide range of response to changes in environmental conditions

**Figure 4:**
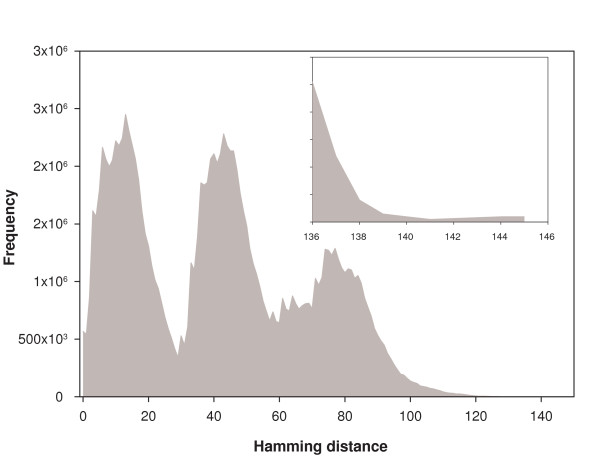
The *E. coli *TRN is flexible in response to changing environmental conditions encountered. Changing the environmental condition can lead to a wide range of hamming distances among the attractors. In the figure, the distribution of pair-wise hamming distances between attractors for 15,427 different environmental conditions is shown. Inset: Enlargement of the graph for large hamming distances. The largest hamming distance obtained between attractors for two different environmental conditions is 145.

While homeostasis is a useful property in any given environmental condition, the organism also needs to respond flexibly to changes in the environment. We investigated flexibility of the TRN to environmental changes in two ways. First, we determined the hamming distance between attractor states of the system 1A corresponding to pairs of minimal media. For the set of 93 minimal media, we found the largest hamming distance between two attractor states corresponding to two different minimal media to be 114. We also determined the attractors of the dynamical system 1A for the larger library of 109732 minimal media (all attractors are fixed points whose basin of attraction is the entire configuration space). We ran 'constrained FBA' for each of these attractors to determine which of them supports a nonzero growth rate (see Methods section for details). This yielded a subset of 15427 minimal media. We computed the pairwise hamming distances among this set of 15427 attractors also. The largest of these distances was found to be 145. The distribution of these hamming distances is trimodal (Fig. 4) similar to that found and discussed in Barrett *et al*. [19]. Thus, although the attractor for a fixed environmental condition is unique, the attractors for two different environmental conditions can be quite far apart. Therefore, while the system is insensitive to fluctuations in gene configurations in a fixed external environment, it can move to quite a different attractor when it encounters a change in environment. Thus the system shows flexibility of response to changing environmental conditions.

**Figure 5:**
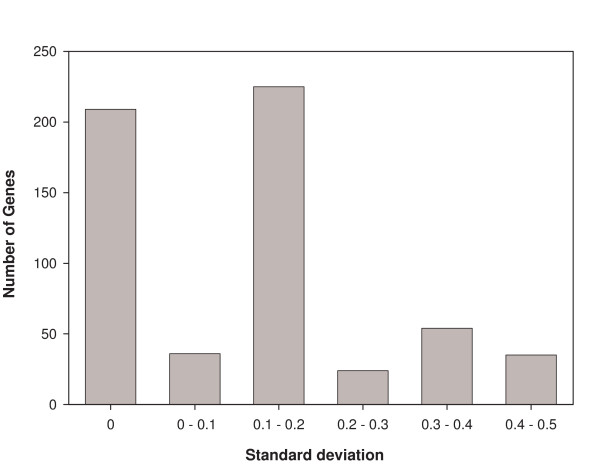
The histogram of standard deviation of a gene's configurations across 15427 attractors for different environmental conditions. The left-most bar corresponds to 209 genes whose configuration remains unchanged.

Second, we found that across these 15427 conditions the genes that had a configuration that differed between any pair of attractors were drawn from a set of 374 out of the 583 genes. Of these 374 genes, 66 genes code for TFs and 308 genes code for metabolic enzymes. The remaining 209 genes had the same configuration (75 off and 134 on) in all the 15427 attractors. The variability of a gene's configuration across different environmental conditions can be characterized by the standard deviation of its value (zero or one) across this set. We found this standard deviation to range from zero to close to its maximum possible value 0.5, with the mean of the 374 standard deviations mentioned above being 0.20. The histogram of standard deviation values is shown in Fig. 5. These observations quantify the considerable variety in a gene's variability across environmental conditions.

### Adaptability: The genetic network's response to changed media increases metabolic efficiency

To further investigate flexibility, we tracked how the metabolic response of the cell, as measured by its growth rate computed using FBA, changes when its environment changes. A reaction in the metabolic network can be assumed to be off if none of the enzymes catalyzing it are being produced, or, equivalently, in our dynamical system, if the genes coding for those enzymes are in the off state. For any configuration of the metabolic genes, FBA can thus be used to compute the growth rate of the cell by turning off all reactions whose corresponding genes are in the off state in that configuration, thereby capturing the effect of gene regulation on metabolic function (see Methods section). We computed this 'constrained FBA' growth rate for each of the attractors of the TRN dynamical system 1A for the 93 minimal media. 81 of them, listed in Table S3 in Additional File 1, gave a nonzero growth rate. Starting from an initial condition of the TRN that corresponds to the attractor of one of these 81 media, say X, we computed the time course of the TRN configuration in another buffered medium Y, until it reached the attractor corresponding to Y. For each of the TRN configurations in the trajectory we computed the growth rate using constrained FBA.

**Figure 6:**
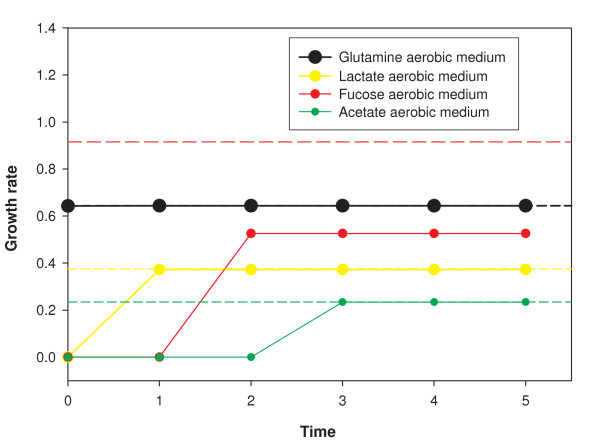
Metabolic efficiency due to regulation. The figure shows the adaptation of the *E. coli *TRN towards higher growth rate in response to change of medium. Growth rate obtained using constrained FBA is plotted for 4 trajectories of the TRN corresponding to aerobic minimal media with glutamine, lactate, fucose or acetate as the carbon source. The initial condition of the TRN in each case is the state the system would have been in for the glutamate aerobic medium. Dotted lines show the pure FBA growth rate in the 4 minimal media. The growth rate increases in three and remains constant in one of these trajectories.

This effectively tracks how the constrained growth rate of the cell changes with time after its environment changes suddenly from X to Y. The result is shown in Fig. 6 for the cases where the carbon source in X is glutamate and in Y is glutamine, lactate, fucose or acetate. In the attractor of X the growth rate is low for the medium Y. The TRN configuration changes with time so as to typically increase the growth rate. We found that for the above 81 minimal media, the growth rate in the attractor configuration of the medium was greater than the average growth rate in the other 80 attractors by a factor of 3.5 (averaged over the 81 media). Moreover the average time to move to the attractor from such initial configurations was only 2.6 time steps. In other words regulatory dynamics enables the cell to adapt to its environment to increase its metabolic efficiency very substantially, fairly quickly.

**Figure 7:**
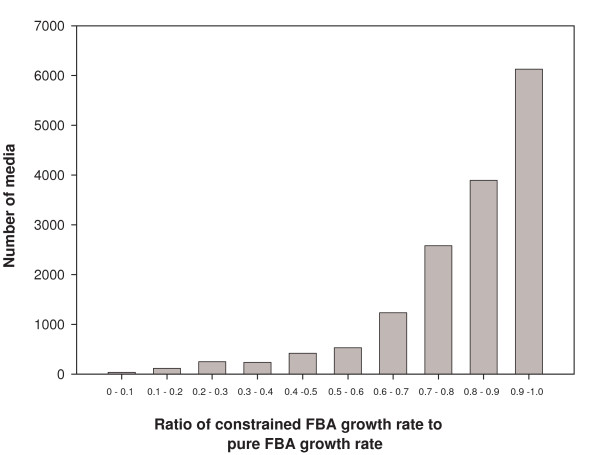
Histogram of the ratio of constrained FBA growth rate in the attractor of each of 15427 minimal media discussed in text to the pure FBA growth rate in that medium. This is peaked in the bin with the largest ratio (≥ 0.9).

We also calculated the growth rate for each of the 15427 minimal media in their respective attractor configurations as a ratio of the maximal growth rate possible in those media (the latter computed for each medium using FBA on the full metabolic network without imposing any regulatory constraints). The average value of this ratio was found to be as high as 0.815 and was less than 0.5 for only 7% of the media (for the histogram of these ratios see Fig. 7). This shows that the regulatory dynamics results in a close-to-optimal metabolic functioning under a large set of conditions. This observation also lends support to the usefulness of FBA in probing metabolic organization.

In a dynamical system of the type *g*_*i*_(*t *+ 1) = *G*_*i*_(**g**(*t*), **m**) it is of course not surprising that the attractor of the genes' configuration **g **depends upon the external metabolite configuration **m**. Our results related to flexibility and adaptability are an attempt to quantify the change in the attractors as the external environment is varied and to show that the change is functionally useful in the survival of the organism.

### Robustness of the network to gene knockouts

**Figure 8:**
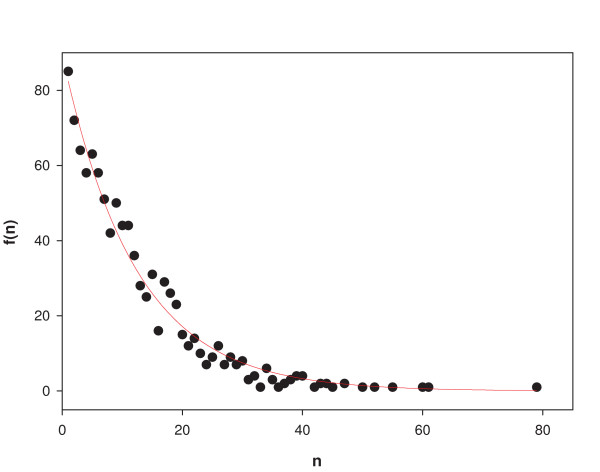
Frequency distribution of the number of random knockouts needed to make a cell unviable for growth for all 81 minimal media. The red curve is the best fit to an exponential distribution.

In order to test the robustness of network functionality to successive gene knockouts, we considered the progressive decline of metabolic performance for an ensemble of 1000 'random knockout trajectories'. Each trajectory was constructed as follows: One out of 583 genes was chosen at random and knocked out, i.e., its *g*_*i *_was set to be identically 0. The constrained FBA growth rate was determined for the attractors of the resultant dynamical system of 582 genes for each of the 81 minimal media discussed above. This was repeated after knocking out another gene chosen at random from the remaining 582 genes, and so on until the attractors for all the 81 media became dysfunctional (i.e., gave a zero growth rate). The number of knockout steps, *n*, needed for the network to become metabolically dysfunctional for all the 81 media was determined for each of the 1000 random knockout trajectories constructed in this way. Figure 8 shows the number or frequency *f*(*n*) of trajectories with a given value of *n*. The curve fits the exponential distribution *f*(*n*) ~ exp(-*n*/*n*_0_) with *n*_0 _= 12.1. Thus the chances of survival decrease exponentially with the number of knockouts.

### Design features of the regulatory network: Origin of homeostasis and flexibility

**Figure 9:**
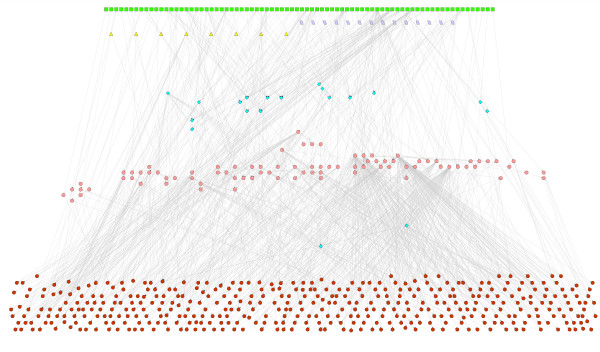
Largest connected cluster of the TRN controlling metabolism in *E. coli*. The colour coding of all nodes is as in Fig. 1.

The following structural characteristics of the TRN explain several of the dynamical features described above: The TRN 1A is an acyclic directed graph with maximal depth 4. The largest connected component is displayed as a hierarchy in Fig. 9, in which all links are pointing downwards. At the bottom of the hierarchy are 479 metabolic genes in the full system (409 in the largest connected component) coding for enzymes that have no outgoing links. Thus these nodes do not influence the dynamics of any other gene. We refer to these as the 'leaves' of the acyclic graph. At the top of the hierarchy are nodes with no incoming links, or 'root nodes'. The depth of a node in the acyclic graph is the length of the longest path to it from a root node. Root nodes correspond to external metabolites and other variables that have fixed values in the system 1A such as certain conditions, fluxes, etc. Since we consider only buffered media the **m **variables, by virtue of their root location, act as control variables of the dynamical system. The genes coding for TFs are at intermediate levels in the graph. These observations immediately explain why (a) there are only fixed point attractors of this system, (b) their basin of attraction is the entire configuration space, (c) it takes at most 4 time steps to reach the attractors from any initial configuration, and (d) the attractor configuration depends upon the medium. For, the **m **vector determines the configuration of the root level. This fixes the configurations of all nodes at the next level (depth 1) at the next time instant (*t *= 1) and subsequent times irrespective of their values at *t *= 0, because the input variables to the Boolean functions controlling them are fixed. This fixes the configurations of all nodes of depth 2 at *t *= 2 irrespective of their configurations at *t *= 1, and so on, until at *t *= 4, the configuration of the maximum depth leaves are fixed irrespective of the configuration they held earlier. A change in the medium or external environment is a change in the configuration of root nodes; this also percolates down in a maximum of 4 steps resulting in a new fixed point. The acyclicity of the *E. coli *TRN was noted by [26]. Its maximum depth being 5 (including parts of the network that regulate systems other than metabolism) was remarked upon by [27]. That root control of this acyclic graph is in the hands of environmental signals has been observed by [28]. However, to our knowledge the present work is the first one that brings these facts together to study dynamics and elaborate upon their consequences for homeostasis and flexibility of the system.

#### Disconnected structure of the reduced dynamical system: modularity, flexibility and evolvability

**Figure 10:**
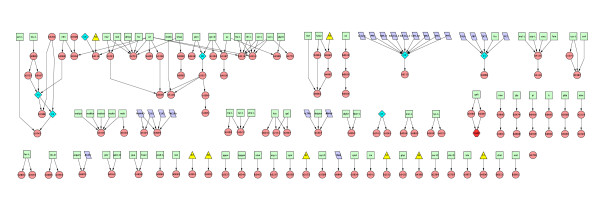
Picture of the regulatory network obtained when all leaf nodes in the network of Fig. 1 are removed along with all their links. The colour coding of all nodes is as in Fig. 1. The red hexagon denotes the lone TF in the network that is coded for by two genes. The nomenclature for conditions C1 to C7 and S1 to S8 is given in Table S4 in Additional File 1. The electronic version of this figure can be zoomed in to read node names.

Since leaf nodes do not affect the dynamics of upstream nodes, it is worthwhile to ask about the dynamics of the 'reduced dynamical system' which is obtained from the full system by removing the leaves. When leaf nodes in the system are removed along with all their links, one is left with Fig. 10. This is a surprisingly disconnected graph; the large connected component has broken up into 38 disconnected components. It has several small components containing upto only 4 nodes at depth ≥ 1 and one component with 27 nodes at depth ≥ 1. The latter component is regulated by oxygen, some inorganic sources of nitrogen, and certain amino acids and sugars. Other components are typically regulated by single metabolites or groups of biochemically related metabolites. This procedure reduces the number of outgoing links from global regulators drastically. For example the gene b3357 coding for Crp is left with only 3 outgoing links instead of 105.

Two components of a dynamical system that are disconnected from each other are dynamically independent: the dynamics of each can be analyzed independently of the other. The dynamics of the 'reduced dynamical system' shown in Fig. 10, in particular its attractors and basins of attraction, can be reconstructed from those of its disconnected components. Such a disconnected or 'product' structure of a dynamical system greatly simplifies its mathematical analysis. Modularity of biological systems refers to the existence of subsystems that are relatively independent of each other [29]. Each connected component of Fig. 10 can therefore be regarded as a core of a module, and modularity of the present genetic regulatory system is then nothing but the property that it is composed of disconnected components at this level of description.

Restoring the leaves and their links in Fig. 10 will take us back to Fig. 1 which contains the large connected component shown in Fig. 9. This means that leaf nodes typically receive links from more than one module core. The structure is like a banyan tree which has multiple trunks emanating from independent roots and in which leaves receive sustenance from more than one root. In this picture, there is no direct crosstalk between the module cores but they can affect common leaves. This enables many leaf nodes to be influenced by several environmental conditions. This 'multitasking' adds to the complexity of cellular response to different environments and possibly contributes to greater metabolic efficiency. When a minimal medium is changed by replacing its carbon source by another that belongs to a different module, the genetic network needs to respond by activating genes coding for enzymes that catalyze metabolic reactions needed to break down the new source and process its moieties. The connections of the leaf nodes to the modules above them must be such that that is achieved, given our finding that the constrained FBA growth rate increases as the new attractor is reached.

The location and dynamical autonomy of the modules could also contribute to evolvability. A new module added to Fig. 10 would not affect existing ones; thus the organism can explore new niches characterized by new food sources without jeopardizing existing capabilities. This may be a particular case of the more general observation [30,31] that the architectural features of organisms responsible for their flexibility to environmental conditions also contribute to their evolvability.

The graph of the dynamical system 1B is not completely acyclic. Effectively some of the genes that are leaves in 1A now get outgoing links that feed back to genes coding for transcription factors. This results in the cycles we have seen as attractors. Our analysis of this dynamical system, not discussed here, reveals that removing the leaves of this system exposes a modular structure in terms of which the attractors can be understood.

#### Almost all input functions are canalyzing in the *E. coli *TRN

It has been shown by Kauffman and his colleagues that the stability in the genetic regulatory networks to perturbations can arise due to the canalyzing property of Boolean functions [23,24]. A canalyzing Boolean function has at least one input such that at least one of the two values of this input determines the output of the function [10]. For a given number of inputs, *K*, the fraction of Boolean functions that are canalyzing decreases as *K *increases. All Boolean rules compiled for eukaryotes from the available literature have been found to be canalyzing functions [21]. For the present *E. coli *TRN the frequency distribution of the number of genes with *K *regulatory inputs is given in Table S5 in Additional File 1. We found that Boolean functions for 579 of the 583 genes in the *E. coli *TRN possess the canalyzing property. Only 4 genes had input functions that were not canalyzing.

#### The dynamical system achieves flexibility even though it is far from the edge of chaos

One might expect that a dynamical system whose attractors have large frozen cores and very small 'twinkling islands' is rather rigid and therefore unlikely to be adaptable to the external environment and also unlikely to be evolvable. This expectation has given rise to the conjecture [10] that genetic regulatory systems ought to be close to the 'edge of chaos', the boundary that separates the ordered phase from the chaotic phase in the space of dynamical systems. However, as discussed above in the section on homeostasis, the present dynamical system is deep in the ordered phase, since it always falls into the same attractor that is a fixed point or has isolated low period cycles for all initial conditions in a few time steps (all or most genes get frozen). In other words it is far from the edge of chaos. We have seen that this is an inevitable consequence of the hierarchical, largely acyclic architecture of the network (see the section on design features). At the same time, we have seen that the system is also highly responsive to the environment. How have these two properties managed to co-exist? The answer lies in the observation that root nodes of the hierarchy are largely the environmental variables – the external metabolites in the present case. The attractor configuration is thus a function of the external environment, specified by the variable **m**. While for any fixed **m **there is a global attractor in which most or all genes have frozen configurations, when **m **changes the genes 'unfreeze' and move to a new attractor configuration. The modular organization of the network with a lot of crosstalk between modules at the leaf level (enzyme coding genes) ensures that the melting and refreezing is quite substantial. The same architecture that produces this flexibility of response to the external environment can also enhance evolvability.

The present architecture as an alternative to the edge of chaos hypothesis for simultaneously producing homeostasis and flexibility has not been noticed earlier because the earlier literature has primarily focussed on the abstract genetic network itself without much reference to the environmental control variables that abound in the real systems. Here, since we are investigating the database iMC1010v1 which brings together, within the same network, genes as well as nodes describing external environmental signals, this possibility has become evident.

## Discussion

All our results, being derived from the database iMC1010v1, have some limitations that stem from the database itself. First, the database covers the regulation of only about half of the metabolic genes in *E. coli*. Even among these genes the present set of connections could have false positives as well as negatives, especially the latter. Additional nodes and connections would modify the dynamics reported here. However, new nodes and connections corresponding to genes coding for enzymes are unlikely to affect our qualitative conclusions about the nature of attractors significantly. The reason is that most such genes are likely to be leaves of the network like the nodes at the bottom of Fig. 9, in which case they would not affect the dynamics of other nodes. However the inclusion of such genes as well as additional connections of existing genes in the network would add to the constraints on FBA; it would be interesting to see the extent to which regulatory dynamics enhances metabolic efficiency in different environmental conditions. The inclusion of more TF genes and modified connections among existing genes would affect the dynamics. In particular feedback loops could bring in longer cycles as attractors. Several genes are known to have autoregulatory self-loops [32] that are not included in the present database. These could produce 2-cycles at the individual nodes even at constant input. Present work seems to indicate that apart from self-loops, TRNs are largely acyclic [26-28] and have a small depth (about 5). Furthermore the kind of modularity described here for the TRN regulating metabolism seems to exist for other parts of the *E. coli *TRN. This together with the evidence of preponderance of canalyzing functions suggests that cyclic attractors where they do exist are likely to be of low period and localized. Cyclicity is needed for explicitly temporal phenomena like the cell cycle or circadian rhythms. It is possible that metabolism being a functionality that needs to be active whenever food is available is largely regulated without cycles at the genetic level, with feedbacks typically entering at the level of metabolites regulating enzymes to ensure efficient functioning on a faster time scale. Nevertheless it would be important to explore these questions with an enlarged database.

In this context, we remark that the lack of feedback from genes to other genes via TFs is not an assumption on our part, rather it reflects the way this biological system actually is as captured in the present database and also in other studies mentioned above. The models studied originally by Kauffman [8,10] were random Boolean networks. Those networks had substantial feedbacks between genes and hence more complicated attractors and dynamics. One of our main results is that the genetic regulatory network of this real biological system is structured (and hence departs from random networks) in such a way that it has simple attractors and dynamics. Thus while our modeling technique is not very different from Kauffman's (apart from the inclusion of the external environment) our dynamical results are quite different because the underlying network has a very different structure from the one Kauffman considered.

In addition to the feedback from genes to other genes via TFs, discussed above, there can be another kind of feedback from the metabolic network (e.g., metabolite concentrations) into the genetic network. The database we have used has such feedbacks via the fluxes of certain metabolic reactions. We have converted these into effective feedbacks from genes to other genes in order to have a simplified dynamics and a closed system of the genes alone (along with external metabolites). We remark that there exist in the literature alternative ways of treating metabolic feedback on regulation and in particular the flux variables. These include the regulatory FBA [11,18,33] and dynamic FBA [34,35] in which the fluxes and the genes are dynamically coupled to each other. However, in these methods one makes an arbitrary choice of the flux vector out of many alternative flux vectors satisfying the constraints. Another method, SR-FBA [25], has been proposed that systematically accounts for multiple optimal metabolic steady states given a regulatory state. However SR-FBA cannot be used for dynamical simulations since it only yields the various steady states for the metabolic-regulatory system. Our treatment of the internal fluxes is simpler compared to the above mentioned methods in that it eliminates the flux variables in favour of an effective feedback of the genes upon other genes. In the context of the present database we believe that our broad conclusions would not change significantly because of our simplified approach to the treatment of the fluxes since the feedbacks from the fluxes affect only 5 genes coding for transcription factors and 16 genes downstream of these coding for enzymes, thus affecting only 21 genes out of 583. A better treatment of the feedbacks from internal metabolites than is achieved by our approach and the other approaches mentioned above requires metabolite concentrations which are difficult to compute at the present time due to the paucity of kinetic information for large scale networks.

We end this section with a comment relating this to earlier works and a speculation. Kauffman [8,10] has found biologically motivated random Boolean networks to possess multiple attractors that he has interpreted as different cell types of a multicellular organism. In the present work, we have studied the genetic network regulating metabolism in a prokaryote. Perhaps not surprisingly, we get a much simpler picture of the network exhibiting a much higher degree of order in the dynamics than the systems Kauffman investigated. While we also find that the system can go into different attractors (see the discussion above on flexibility), yet, unlike Kauffman, for whom different attractors were realized via different initial conditions of the genes, in the present case the different attractors are realized when the control variables (metabolites in the external environment) have different configurations. When the control variables are held fixed we find no (or very little) multiplicity of attractors irrespective of the initial condition of the genes (see the discussion on homeostasis). The architecture and dynamics we have found is probably quite suitable for prokaryotic lifestyles and evolution. The question remains open whether for eukaryotes and especially multicellular ones, the hypothesis that associates different cell types with different attractors of the regulatory dynamics is valid. While that hypothesis remains an enticing possibility, it is worth noting that the present simple architecture would have its uses in the eukaryotic case as well. Environmental control of cellular attractors (via the architecture discussed above) can itself cause a cell to differentiate into another type, the environment being determined in the multicellular case by the state of other cells in the organism. The modular structure discussed above would even permit a cell to hop through several attractors in the course of development of the organism as the environmental cues to this cell change. Such an architecture could thus contribute to developmental flexibility and, potentially, evolvability of eukaryotes as well. The multiplicity of internal attractor basins pointed out by Kauffman would be an asset in keeping the cell in its new attractor after a transient environmental cue has caused it to shift from one basin to another. It would be interesting to investigate these questions when a database similar to iMC1010v1 becomes available for a multicellular organism.

## Conclusion

The overall organizational picture of the system that emerges from our study is the following: The genetic regulatory network controlling metabolism in *E. coli *as represented in the present database is (essentially) an acyclic graph. Cycles, where they do exist, are short and 'localized' in that they have a limited number of nodes downstream of them in the present system. The nodes at the root of this graph are primarily environmental variables (in the present case external metabolites). The leaves of this graph are genes coding for enzymes while the middle layers correspond to genes coding for transcription factors. The maximal distance between a root node and a leaf node is 4. The top and middle layers are organized into small dynamically independent modules; crosstalk between the modules occurs at the lower level of enzyme coding genes. The database, and hence the above mentioned architecture, represents a limited portion of the genetic regulatory network of *E. coli *and can change as more information becomes available. Nevertheless, this architecture has the virtue that it endows the system with the property of homeostasis, namely, that in a fixed environment the genes relax within a short time to the same fixed configuration after being perturbed. It is also responsible for the property that the system responds in a flexible and efficient way to a sustained change of environment. We have speculated that such an architecture can contribute to the evolvability of the network, and variants of it might be useful for multicellular organisms as well.

## Methods

### Construction of the Boolean dynamical system describing the genetic regulation of *E. coli *metabolism

We have represented the *E. coli *TRN regulating its metabolism as a Boolean dynamical system given by the equation (1) where *g*_*i*_(*t*) represents the configuration of gene *i *(with values 0 or 1 representing the gene being off or on, respectively) at time *t*, and the vector **m **= (*m*_1_,..., *m*_96_) describes the buffered external environment (*m*_*i *_being 0 or 1 if metabolite *i *is absent or present, respectively, in the external environment). This dynamical system was constructed from the integrated regulatory and metabolic network iMC1010v1 for *E. coli *[11]. This database was downloaded from the website [36]. The regulatory interactions and the Boolean rules incorporated in this reconstructed network are based on various literature sources. The TRN accounts for 583 genes of which 479 are coding for enzymes catalyzing metabolic reactions and 104 are coding for TFs. The 583 genes, 103 TFs, 96 external metabolites, 19 conditions, and 21 internal fluxes of metabolic reactions are respectively denoted by the vectors **g**, **t**, **m**, **c**, **v**, all of which can, in principle, depend upon time *t*. E.g., *t*_*i*_(*t*) (*i *= 1, 2,...,103), the *i*^*th *^component of **t**(*t*), equals unity if the TF *i *is present in the cell at time *t *and zero if not. *c*_*i*_(*t*) (*i *= 1,2,...,19), the *i*^*th *^component of **c**(*t*), equals unity if the *i*^*th *^condition holds at time *t *and zero if not. *v*_*i*_(*t*) (*i *= 1, 2,...,21), the *i*^*th *^component of **v**(*t*), equals unity if the *i*^*th *^metabolic reaction in the above mentioned set of internal metabolic reactions is happening inside the cell at time *t *(with a flux greater than a specified value) and zero if not. The additional 9 stimuli (e.g. stress, etc.) are assumed to be absent. Thus the overall system contains 583+103+96+19+21 = 823 Boolean variables. Its dynamics is organized as follows: The presence or absence of the transcription factors, external metabolites, and the status of the internal fluxes and other conditions at time *t *determines the on-off state of the 583 genes at *t*:

*g*_*i*_(*t*) = *F*_*i*_(**t**(*t*), **m**(*t*), **c**(*t*), **v**(*t*)),   *i *= 1, 2,...,583.

The database iMC1010v1 gives the form of the functions *F*_*i *_in terms of AND, OR and NOT operations on the Boolean arguments. The 103 transcription factors are coded for by a subset of 104 genes (two genes together code for one TF and the remaining 102 genes code for one TF each). The on-off state of these genes at the previous time step *t *- 1 determines whether the TFs they code for are present at *t *(a single time step therefore corresponds to the average time for transcription and translation). Thus

*t*_*i*_(*t*) = *T*_*i*_(**g**(*t *- 1)),   *i *= 1, 2,...,103,

where the function *T*_*i*_(**g**) = *g*_*i *_for 102 transcription factors that are coded for by single genes; for the TF coded for by 2 genes *T*_*i*_(**g**) = $g_{i_{1}}$*AND *$g_{i_{2}}$. Substituting this in the previous equation gives

*g*_*i*_(*t*) = *F*_*i*_(**T**(**g**(*t *- 1)), **m**(*t*), **c**(*t*), **v**(*t*)).

This equation provides the dynamical rule for updating the gene configurations from one instant to the next, provided the status of the variables **m**, **c**, **v **is known.

### Treatment of external metabolites m

In this work we considered only buffered media in which the external environment was assumed constant. Thus **m**(*t*) = **m**, independent of *t*. For each medium considered, the components of **m **corresponding to the metabolites present in the external environment were set to unity and the remaining components were set to zero. *E. coli *is known to be capable of transporting 143 metabolites into the cell, including 131 organic and 12 inorganic molecules [37] of which 96 (86 organic and 10 inorganic) are included in the regulatory part of the database iMC1010v1. We considered the following classes of minimal media in this work:

(a) 93 minimal media (61 aerobic and 32 anaerobic): These are characterized by a single organic source of carbon (listed in Supplementary Table S1 in Additional File 1), and the ions of ammonium, sulphate, phosphate, hydrogen, iron, potassium and sodium. The components of **m **corresponding to these metabolites were set to unity and others were set to zero in a given minimal medium. Oxygen was set to unity in the aerobic media and to zero in anaerobic media. In principle 86 organic carbon sources would yield 172 media (aerobic plus anaerobic). Out of these we restricted ourselves to that subset of media for which the *E. coli *metabolic network supports growth of the cell as determined by Flux Balance Analysis (FBA); i.e., media for which the optimal growth rate calculated by FBA without imposing regulatory constraints is nonzero (see below). This condition yielded the list of 93 media listed in Supplementary Table S1 in Additional File 1. Most of the work reported in this paper was performed with this set of minimal media.

(b) For part of our work we also considered a much larger library of minimal media, described by [19], in which all possible combinations of single sources of carbon, nitrogen, sulphur, phosphorus, etc., from among the 143 metabolites ingested by *E. coli *are considered. Following the method described in the supplementary material of [19] gave us a library of 109732 minimal media.

### Treatment of the conditions c and internal fluxes v

*The ***c ***variables: *Of the 19 Boolean variables *c*_*i*_(*t*), 15 depend only on the configuration of a subset of TFs and external metabolites at time *t*, i.e., *c*_*i*_(*t*) = *C*_*i*_(**t**(*t*), **m**(*t*)), *i *= 1, 2,...,15, where the *C*_*i *_are specified Boolean functions in the database. These functions can be substituted in Eq. 2. This eliminates these 15 variables *c*_*i *_from the dynamical system at the expense of a more complicated effective dependence of *g*_*i*_(*t*) on **t**(*t*) and **m**. Of the remaining 4 conditions, one, representing growth of the cell, is set to unity (since we primarily consider only those conditions in which the cell has a nonzero growth rate). Another condition represents the pH of the external environment, which we take to be between 5.5 and 7 (weakly acidic, as, for example, in the human gut). The pH condition affects only 3 genes in the database. For two of them the operative regulatory clause is 'pH < 4'; we take the Boolean variable *c*_*i *_corresponding to pH to be zero (false) for these two genes. For the third gene the clause is 'pH < 7'; for this gene we take this variable to be unity (true). Two other conditions, designated as 'surplus FDP' and 'surplus PYR' in the database, correspond to whether 'surplus' amounts of fructose 1,6-bisphosphate and pyruvate are being produced in the cell. These conditions depend upon the values of some of the internal fluxes *v*_*i *_and the presence of an external metabolite, fructose, through specified Boolean functions. The latter variable is treated as unity if the minimal medium includes fructose and zero otherwise, as discussed above. The treatment of the internal fluxes is discussed below.

*The ***v ***variables: *The 21 components of the vector **v **represent fluxes of 21 metabolic reactions. As mentioned by [11], these are surrogate for other conditions inside the cell, e.g., concentrations of metabolites produced by those reactions, which can affect gene regulation. We have treated these variables in two distinct ways.

(A) In the first approach we identified whether the particular metabolic reaction was a 'blocked reaction' or not [38-40]. A reaction is said to be blocked in a particular environmental condition (specified by a buffered medium) if under that medium no steady-state flux is possible through it [39]. This can be determined using metabolic flux analysis methods from a knowledge of the metabolic network. For each medium (specified by the vector **m**) we chose the fixed value zero for a particular flux variable *v*_*i*_(*t*) if it was found to be blocked for that condition, and unity otherwise. Thus in this approach the *v*_*i *_were not dynamical variables, but rather fixed parameters (albeit fixed with an eye on self-consistency).

(B) In the second approach, we allowed the *v*_*i *_to be dynamical, but made a simplifying assumption about their dynamics. In the cell, the flux values of individual reactions are determined by the concentrations of participating metabolites and the catalyzing enzymes, the latter being controlled by the activity of their respective genes. In a discrete-time approximation, an enzyme is present at time *t *if the genes coding for it are active at *t *- 1. Thus we set *v*_*i*_(*t*) = 1 if the genes coding for the enzyme of that metabolic reaction were active at *t *- 1, and *v*_*i*_(*t*) = 0 otherwise. This could be done for a subset of 10 out of 21 reactions, since the genes of their enzymes were part of the 583 genes in the database. Genes coding for the enzymes of the remaining 11 reactions were not part of the database and hence the corresponding *v*_*i *_could not be made dynamical variables in this fashion. These latter *v*_*i *_were fixed as in part (A) for each medium. The approach (B) introduces feedbacks in the genetic regulatory network.

Our above treatment defines the substitutions to be made in Eq. 2 for the variables **c**(*t*), **v**(*t*). Each component of **c **in Eq. 2 is either a specified Boolean function of **t**(*t*), **m**, and **v**(*t*), or is a suitably chosen Boolean constant. Each component of **v**(*t*) is, in turn, either a specified Boolean function of **g**(*t *- 1), or is a suitably chosen Boolean constant. These substitutions together with Eq. 3 make the right hand side of Eq. 2 a function of only **g**(*t *- 1) and **m**, i.e., Eq. 2 reduces to *g*_*i*_(*t*) = *G*_*i*_(**g**(*t *- 1), **m**), which is the same as Eq. 1. The functions *G*_*i *_define the final dynamical system, and include information coming from the functions *F*_*i*_, as well as the dependence of **t**, **c **and **v **on **g **and **m**. Note that the choices (A) and (B) for the **v **variables yield different dynamical systems for Eq. 1 which we denote as 1A and 1B respectively; in 1B 6 out of 583 genes have additional links from other genes in the set compared to 1A. Programs implementing these two dynamical systems are available from the authors.

### Computation of Growth rate of *E. coli *for a given environmental condition

Flux Balance Analysis (FBA) is a computational technique that determines the maximal steady state growth rate of a cell that its metabolic network can support in any given buffered medium [4,5,7]. The database iMC1010v1 [11] includes the *E. coli *metabolic network database iJR904 [37] to which FBA can be applied. In this work we use FBA in two ways:

*Pure (unconstrained) FBA*. This uses the full metabolic network iJR904 (without any constraints from regulation) to calculate the maximal growth rate of the cell under various media. A zero value of the maximal growth rate for a particular medium means that the metabolic network does not contain pathways to convert the substances present in the medium into 'biomass metabolites' needed for cell growth. 

*FBA with regulatory constraints*. Of the 583 genes in the database iMC1010v1 479 genes code for enzymes of the metabolic reactions in the database iJR904. In any given configuration of the genetic network a subset of these genes is off and the remaining are on. Thus one can run FBA wherein those reactions of the metabolic network are switched off whose enzymes are not being produced (i.e., whose corresponding genes are off). We will refer to this as 'constrained FBA'. In this way one can track the optimal growth rate as a function of time as the configuration of the genes changes according to the dynamics of the genetic regulatory network, as discussed in [11,18]. The growth rate obtained from constrained FBA for any configuration of the genes is, by definition, less than or equal to that obtained from pure FBA (for the same medium).

## Authors' contributions

SJ conceived the research project. AS and SJ designed the research. AS performed the simulations. Both authors analyzed the data, wrote the paper and approved the final manuscript.

## Supplementary Material

Additional file 1 Supplementary Tables S1 to S5. Click here for file
