# Supplementary material for: The regulatory network of E. coli metabolism as a Boolean dynamical system exhibits both homeostasis and flexibility of response
Source: BMC Syst Biol. 2008 Feb 29;2:21. doi: 10.1186/1752-0509-2-21 (PMC2322946; doi:10.1186/1752-0509-2-21)
Supplement: Additional file 1 — Supplementary Tables S1 to S5. [file 1752-0509-2-21-S1.pdf]

Additional File 1: Supplementary Tables

Table S1: List of minimal media considered as environmental conditions to study *E. coli* TRN. The 62 minimal media listed here are considered in aerobic conditions. The first 31 media shaded in grey are considered also in anaerobic conditions. Each carbon source is provided along with ammonia, sulphate, phosphate, proton, iron, potassium and sodium for uptake. Oxygen is provided in aerobic conditions. See supporting text (section on Treatment of external metabolites **m**) for a discussion on how this list was compiled.

Serial number	Carobon Source	Abbreviation of the carbon source
1	2-Dehydro-3-deoxy-D-gluconate	2ddglcn
2	N-acetyl-D-glucosamine	acgam
3	L-Arabinose	arab-L
4	Cytidine	cytd
5	D-Fructose	fru
6	L-Fucose	fuc-L
7	D-Glucose 6-phosphate	g6p
8	D-Galactose	gal
9	D-Galactarate	galct-D
10	D-Galactonate	galctn-D
11	Galactitol	galt
12	D-Glucosamine	gam
13	D-Glucose	glc-D
14	D-Gluconate	glcn
15	D-Glucarate	glcr
16	L-idonate	idon-L
17	Inosine	ins
18	Lactose	lcts
19	Maltose	malt
20	Maltohexaose	malthx
21	Maltopentaose	maltpt
22	Maltotriose	malttr
23	Maltotetraose	maltttr
24	D-Mannose	man
25	Melibiose	melib
26	D-Ribose	rib-D
27	L-Rhamnose	rmn
28	D-Sorbitol	sbt-D
29	Trehalose	tre
30	Xanthosine	xtsn
31	D-Xylose	xyl-D
32	3-(3-hydroxy-phenyl)propionate	3hpppn
33	Acetate	ac
34	Acetoacetate	acac
35	D-Alanine	ala-D
36	L-Alanine	ala-L
37	L-Arginine	arg-L
38	L-Asparagine	asn-L
39	L-Asparate	asp-L
40	Citrate	cit
41	Fumarate	fum
42	L-Glutamine	gln-L
43	L-Glutamate	glu-L
44	Glycine	gly
45	Glycerol	glyc
46	Glycolate	glyclt
47	Hexadecanoate (n-C16:0)	hdca

48	D-Lactate	lac-D
49	L-Lactate	lac-L
50	L-Malate	mal-L
51	D-Mannitol	mnl
52	Octadecanoate (n-C18:0)	ocdca
53	Phenylpropanoate	pppn
54	L-Proline	pro-L
55	Pyruvate	pyr
56	D-Serine	ser-D
57	L-Serine	ser-L
58	Succinate	succ
59	L-tartrate	tartr-L
60	L-Threonine	thr-L
61	L-Tryptophan	trp-L
62	Tetradecanoate (n-C14:0)	ttdca

Table S2: List of 21 genes whose configuration can oscillate

Gene	bnumber
nagC	b0676
nagA	b0677
nagB	b0678
nagE	b0679
deoR	b0840
uxaB	b1521
kdgR	b1827
uxaA	b3091
uxaC	b3092
exuT	b3093
exuR	b3094
kdgK	b3526
glmU	b3730
kdgT	b3909
uxuA	b4322
uxuB	b4323
uxuR	b4324
deoC	b4381
deoA	b4382
deoB	b4383
deoD	b4384

Table S3: Comparison of growth rate obtained using pure (unconstrained) FBA with that obtained using constrained FBA for various minimal media (see main text, methods section). For each media, the amount of carbon source uptake was set to 10 mM per g-DCW per hr and the uptake rates of all other inorganics in the media was left unconstrained.

Serial Number	Minimal Media	Oxygen Availability	Growth Rate with regulatory constraints (GRreg)	Growth Rate with no regulatory constraints (GRpure)	Ratio (GRreg/GRpure)
1	ac	aerobic	0.234	0.234	0.998
2	ala-D	aerobic	0.416	0.423	0.985
3	ala-L	aerobic	0.416	0.423	0.985
4	arab-L	anaerobic	0.785	0.786	0.998
5	arab-L	aerobic	0.220	0.222	0.992
6	arg-L	aerobic	0.743	0.784	0.948
7	asn-L	aerobic	0.452	0.452	0.999
8	asp-L	aerobic	0.451	0.451	0.998
9	cytd	anaerobic	0.826	0.872	0.948
10	cytd	aerobic	0.282	0.394	0.716
11	ddglcn	anaerobic	0.830	0.831	0.998
12	ddglcn	aerobic	0.226	0.228	0.993
13	fru	anaerobic	0.955	0.957	0.998
14	fru	aerobic	0.297	0.299	0.992
15	fuc-L	aerobic	0.526	0.915	0.575
16	fuc-L	anaerobic	0.156	0.158	0.993
17	fum	aerobic	0.439	0.439	0.998
18	g6p	anaerobic	0.990	0.992	0.998
19	g6p	aerobic	0.377	0.380	0.992
20	gal	anaerobic	0.944	0.946	0.998
21	gal	aerobic	0.270	0.272	0.992
22	galct-D	anaerobic	0.663	0.664	0.998
23	galct-D	aerobic	0.209	0.210	0.993
24	galctn-D	anaerobic	0.830	0.831	0.998
25	galctn-D	aerobic	0.226	0.228	0.993
26	galt	anaerobic	1.007	1.009	0.998
27	galt	aerobic	0.253	0.255	0.993
28	gam	anaerobic	0.955	0.957	0.998
29	gam	aerobic	0.297	0.299	0.992
30	glc-D	anaerobic	0.955	0.957	0.998
31	glc-D	aerobic	0.297	0.299	0.992
32	glcn	anaerobic	0.876	0.877	0.998
33	glcn	aerobic	0.241	0.243	0.990
34	glcr	anaerobic	0.663	0.664	0.998
35	glcr	aerobic	0.209	0.210	0.993
36	gln-L	aerobic	0.644	0.644	0.999
37	glu-L	aerobic	0.670	0.674	0.994
38	glyc	aerobic	0.555	0.555	0.998
39	glyclt	aerobic	0.177	0.177	0.998
40	hpppn	aerobic	1.124	1.125	0.999
41	idon-L	anaerobic	0.866	0.867	0.998
42	idon-L	aerobic	0.207	0.208	0.992
43	ins	anaerobic	0.888	0.889	0.998
44	ins	aerobic	0.350	0.352	0.995
45	lac-D	aerobic	0.410	0.413	0.992
46	lac-L	aerobic	0.372	0.375	0.992

47	lcts	anaerobic	1.900	1.903	0.998
48	lcts	aerobic	0.566	0.571	0.992
49	mal-L	aerobic	0.427	0.439	0.971
50	malt	anaerobic	1.911	1.914	0.998
51	malt	aerobic	0.593	0.598	0.992
52	malthx	anaerobic	5.826	5.835	0.998
53	malthx	aerobic	1.995	2.010	0.992
54	maltpt	anaerobic	4.824	4.832	0.998
55	maltpt	aerobic	1.591	1.603	0.992
56	malttr	anaerobic	2.867	2.871	0.998
57	malttr	aerobic	0.890	0.897	0.992
58	maltttr	anaerobic	3.822	3.828	0.998
59	maltttr	aerobic	1.186	1.195	0.992
60	man	anaerobic	0.955	0.957	0.998
61	man	aerobic	0.297	0.299	0.992
62	melib	anaerobic	1.900	1.903	0.998
63	melib	aerobic	0.566	0.571	0.992
64	mnl	aerobic	1.020	1.025	0.995
65	pro-L	aerobic	0.754	0.762	0.990
66	pyr	aerobic	0.346	0.348	0.992
67	rib-D	anaerobic	0.750	0.751	0.998
68	rib-D	aerobic	0.139	0.140	0.992
69	rmn	aerobic	0.526	0.915	0.575
70	rmn	anaerobic	0.156	0.158	0.993
71	sbt-D	anaerobic	1.020	1.025	0.995
72	sbt-D	aerobic	0.256	0.258	0.992
73	ser-D	aerobic	0.346	0.348	0.992
74	ser-L	aerobic	0.354	0.356	0.994
75	succ	aerobic	0.469	0.469	0.998
76	tre	anaerobic	1.911	1.914	0.998
77	tre	aerobic	0.593	0.598	0.992
78	xtsn	anaerobic	0.857	0.859	0.998
79	xtsn	aerobic	0.346	0.348	0.995
80	xyl-D	anaerobic	0.785	0.786	0.998
81	xyl-D	aerobic	0.220	0.222	0.992

Table S4: Abbreviations used to label nodes corresponding to conditions and stimuli in Fig. 4 and their corresponding names

Abbreviation	Name
C1	CRP noGLC
C2	Surplus FDP
C3	Surplus PYR
C4	NRI_low
C5	NRI_hi
C6	Growth
C7	pH
S1	Dipyridyl
S2	High NAD
S3	Heat shock
S4	Stress
S5	Oxidative stress
S6	LBMedia
S7	High osmolarity
S8	Salicylate

Table S5: The table shows the number of genes in *E. coli* TRN with K regulatory inputs

Number of regulatory inputs K	Number of Genes
1	259
2	189
3	68
4	39
5	10
6	4
8	2
